# Esthetic Management of an Anterior Avulsed Tooth: A Case Report

**DOI:** 10.5005/jp-journals-10005-1016

**Published:** 2009-12-26

**Authors:** Prabhakar AR, Sugandhan, Roopa KB, Akanksha Gulati

**Affiliations:** 1 Professor and Head, Department of Pedodontics and Preventive Dentistry, Bapuji Dental College and Hospital Davangere-577004, Karnataka, India; 2 Professor, Department of Pedodontics and Preventive Dentistry, Bapuji Dental College and Hospital, Davangere-577004 Karnataka, India; 3 Postgraduate Student, Department of Pedodontics and Preventive Dentistry, Bapuji Dental College and Hospital Davangere-577004, Karnataka, India; 4 Postgraduate Student, Department of Pedodontics and Preventive Dentistry, Bapuji Dental College and Hospital Davangere-577004, Karnataka, India

**Keywords:** Avulsion, anterior tooth, esthetic, semi-permanent.

## Abstract

Avulsion and luxation account for up to 16% of all traumatic injuries in the permanent dentition and 7.2% of injuries in the primary dentition. A range of treatment options are available that can help conserve the tooth after a traumatic episode. There are, however, occasions where loss of the traumatized tooth is inevitable with special regard to avulsion injuries. replantation of teeth having doubtful long-term prognosis. Following the traumatic loss of an anterior tooth it is important that an immediate replacement is provided in order to avoid esthetic, masticatory and phonetic difficulties and to maintain the edentulous space to avoid arch length discrepancy. The loss of an anterior tooth in a child or young adolescent may present a difficult prosthetic problem.^3^ This problem can be managed in several ways. This article reports utilization of the avulsed tooth as part of fixed semi-permanent bridge.

## INTRODUCTION

Avulsion and luxation are complex injuries that affect multiple tissues, accounting up to 16% of all traumatic injuries in the permanent dentition and 7.2% of injuries in the primary dentition.^1^ According to the world health organization (WHO) classification of dental injuries, avulsion is the complete displacement of the tooth from its alveolar socket. In the permanent dentition, the maxillary central incisor is the mostfrequently avulsed and luxated tooth. Sport and automobile accidents are common causes, involving mainly children, aged 7-10 years old. The loss of solitary permanent maxillary tooth in a child or young adolescent is usually because of either direct trauma or its sequel.

The large number of motor vehicle accident injuries with multiorgan trauma, the necessity for life saving and lack of dental instrument in the emergency and operating rooms, may lead the general medical practitioner or surgeon to ignore the dental trauma or postpone dental treatment to later stage.

The most important factor in the treatment of injured teeth is time. The longer the time lapse between tooth avulsion and reimplantation, greater the risk of replacement resorption and inflammatory root resorption. There is a range of treatment options that can be provided to conserve the tooth after a traumatic episode. There are occasions where loss of the traumatized tooth is inevitable with special regard to avulsion injuries, replantation of teeth has doubtful long term prognosis. Because of resorption more than half of the teeth are eventually lost because of ankylosis or inflammatory resorption. Following the traumatic loss of an anterior tooth it is important that an immediate replacement is provided in order to avoid esthetic, masticatory and phonetic difficulties and to maintain the edentulous space to avoid arch length discrepancy.^2^ This article reports utilization of the avulsed tooth as part of fixed semi-permanent bridge.

## CASE REPORT

**Fig. 1.**
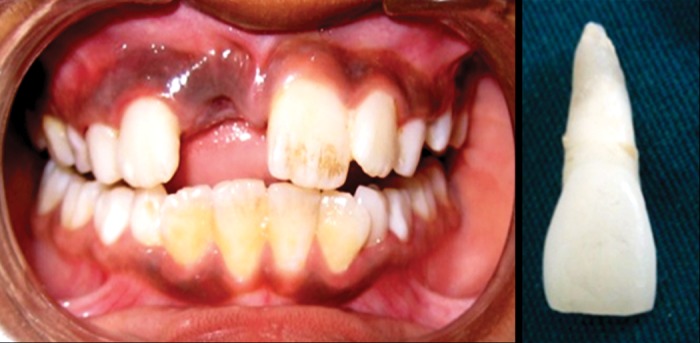
Intraoral view and avulsed maxillary permanent right central incisor

An 11 years old boy reported to the Department of Pedodontics and Preventive Dentistry, Bapuji Dental College and Hospital with a chief complain of a dislodged tooth. The maxillary permanent right central incisor had been avulsed two days ago (Fig. 1). The trauma took place in school when the child slipped on the staircase and hit his mouth. The child remained conscious and there was no history of vomiting, or bleeding from the nose or ears after the injury. The parents failed to report to the dental clinic for 2 days after the injury. They had however stored the tooth in milk in a plastic jar. The child’s health history was noncontributory; he was not taking any medications, and had no known drug allergies or systemic illness. The status of his tetanus immunization was unknown.

Extraoral examination revealed no soft tissue injury, asymmetry of the head and neck region, or cervical lymphadenopathy. Intraoral examination revealed no soft tissue injury. The alveolar socket of the avulsed maxillary permanent right central incisor appeared intact. Intraoral periapical radiograph of the area showed no remaining tooth particles or debris in the socket. No injury to the adjacent teeth was found. On examination of the avulsed tooth the crown and root appeared intact with no visible signs of damage. Root completion of the tooth had taken place.

As the extraoral time of the avulsed tooth was about 48 hours, the decision was made to avoid replanting the tooth. Because both the patient and his parents were concerned with aesthetics, the possibility of using the clinical crown as part of a fixed appliance was proposed. Upon approval of the patient and parents treatment was initiated.

The avulsed tooth was rinsed in normal saline, and the tooth was sectioned in a horizontal plane at the level of the cement-enamel junction with the help of an abrasive disk under constant irrigation. Complete extirpation of the pulp was done from the cervical area. The crown portion was then contoured with a flame-shaped air-rotor bur. The entire pulp chamber was etched with 37% phosphoric acid, followed by rinsing for 15 seconds. An acetone-based single bottle adhesive (Prime and Bond NT, Dentsply, Konstanz, Germany) was applied to the etched surface in accordance with manufacturers recommendations. Thereafter a hybrid resin composite material (Z100, 3M ESPE, St. Paul, MN) was placed in increments and light cured for 40 seconds. The tooth was then held in its anatomical position in the mouth in passive contact with the socket, and checked for appropriateness of size and occlusion.

**Fig. 2.**
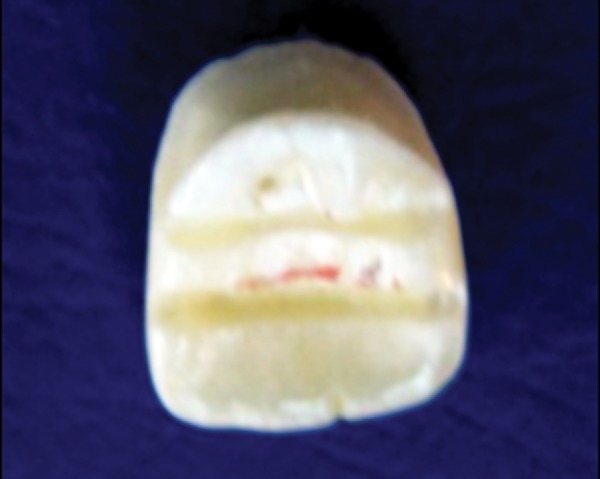
Palatal surface of the prepared crown with groove in the middle third

**Fig. 3.**
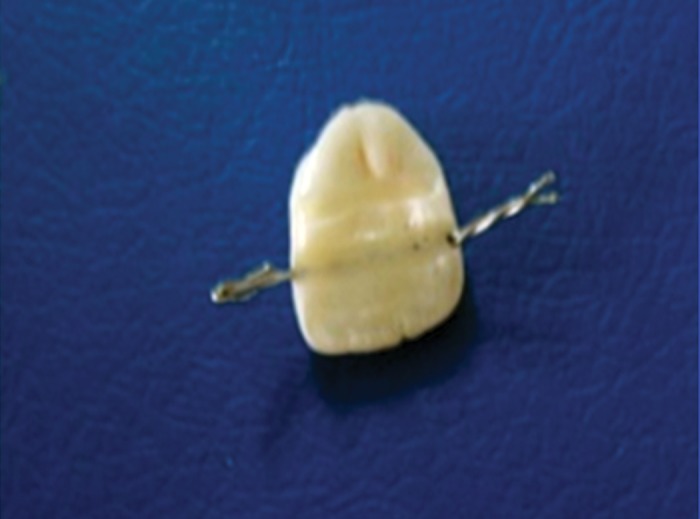
Wire fixed into the groove with light curing composite resin

**Fig. 4.**
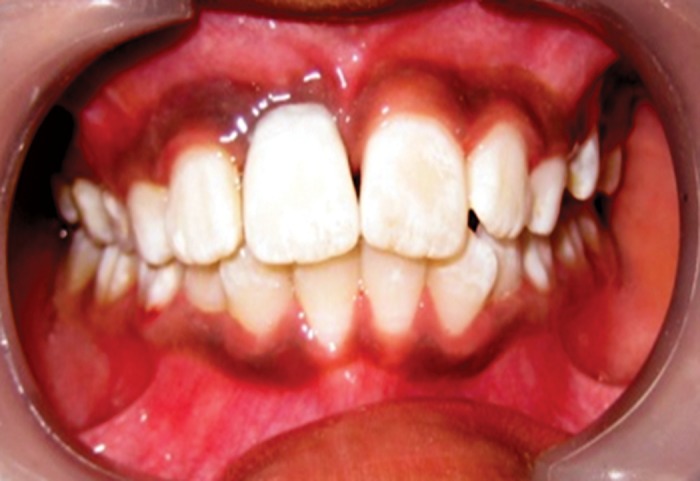
Tooth and wire component fixed in position with light curing composite resin

**Fig. 5.**
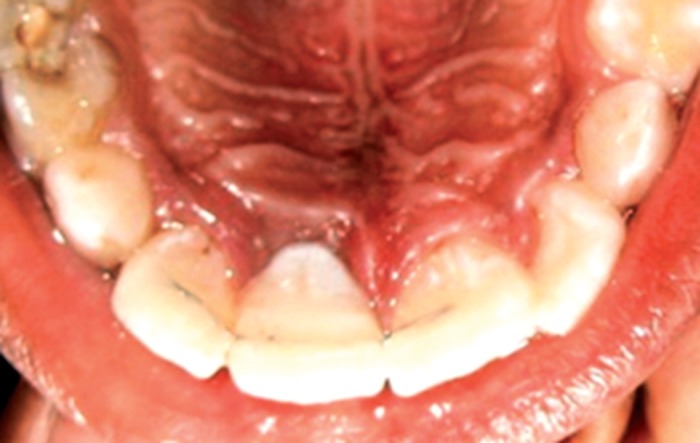
Tooth with wire fixed in position with light curing composite resin-palatal view

The mesiodistal widths of the right and left central incisor, and the right lateral incisor were measured. Three strands of 0.010 inch orthodontic ligature wire were twisted together and one piece of the measured length was cut. With the help of a straight fissure air-rotor bur a groove were made onto the palatal surface of the prepared crown in the middle third, ensuring it would not interfere with the child’s occlusion (Fig. 2). The ligature wire was then fixed into the groove with light curing composite resin (Z100, 3M ESPE, St. Paul, MN) (Fig. 3). The crown was then placed in its desired position and the wire to be fixed onto the adjacent teeth was adapted passively to their lingual surface with the help of a plier. They were then fixed in position with light curing composite resin (Figs 4 and 5). The occlusion was once again checked for appropriateness during excursive movements.

Both the patient and the parents were given oral hygiene instructions. He was placed on a soft diet for one week, and told to avoid biting from the front two teeth. The first recall appointment was made one week later.

## DISCUSSION

The loss of an anterior tooth in a child or young adolescent may present a difficult prosthetic problem.^3^ This problem can be managed in several ways, including (1) a provisional removable partial denture replacing the missing tooth (teeth); (2) a provisional fixed acrylic bridge utilizing the adjacent teeth as full crown abutments; or (3) a bonded bridge using either a denture tooth, or a chairside fabricated composite resin tooth as the tooth replacement.^4^

These techniques have their advantages and disadvantages when fabricating an aesthetically acceptable result. The disadvantage of a removable partial denture is that the acrylic denture base is bulky and must cover the soft tissue. In many cases adjusting to this restoration is difficult for the young patient. In the case of a conventional full-coverage crown retained fixed partial denture, a major disadvantage of the procedure is that it may involve the preparation of healthy abutment teeth for crowns. When it involves a young child, the anatomic considerations of size of the pulp, continuing soft tissue changes as the teeth continue to erupt, and other growth and development that will occur preclude the use of crown preparations.

The technique described in this article has several advantages over other methods for the immediate replacement of an anterior tooth that has been avulsed.^5^ These are as follows:

- The clinical skills and material needed for the procedure are easily available in all dental clinics.
- The effect of tooth loss is better tolerated by the patient since immediate replacement, with the natural tooth is achieved.
- It avoids a laboratory stage, which reduces the cost and the number of treatment visits.
- Use of the natural tooth ensures consistency of shape and shade which is not always easy to achieve in a fixed/ removable prosthesis.
- Does not require preparation of the abutment teeth.
- Excessive post-traumatic ridge resorption can be managed by the addition of further composite resin or glass ionomer cement to the fitting surface of the tooth.
- The patient avoids the difficulties of acclimatization with a removable appliance.

Implants are the treatment of choice and should be considered when general and local conditions are favorable. Their use is generally not intended before the end of the growth period and around the age of 18 years. Because of their high cost, poor financial condition could also limit their use. More economically acceptable treatments should therefore be investigated for the replacement of a missing tooth, as a main treatment or as a long-term provisional treatment before implant therapy.^6^

## CONCLUSION

The technique described in this article suggests a new treatment option for the replacement of a missing anterior tooth. This technique restores aesthetics and function. It is more comfortable for the young patient than a removable appliance. It does not require any tooth reduction and can be easily modified or repaired by simple chair-side procedures, until the time a more definitive restoration can be placed. The noninvasive and expedient nature of this technique made it superior to all other options.

## References

[1] Gabris K, Tarjan I, Rozsa N (2001). Dental trauma in children presenting
for treatment at the Department of Dentistry for Children and
Orthodontics, Budapest, 1985-1999. Dent Traumatol.

[2] Ulusoy AT, Cehreli ZC (2008). Provisional use of a natural
tooth crown following failure of replantation: A case report. Dent Traumatol.

[3] Daly CG, Wilkinson EJ (1983). Use of patient’s natural crown as the
pontic in a composite resin-retained temporary bridge. Aust Dent J.

[4] Strassler HE (1995). Aesthetic management of traumatized anterior
teeth. Dent Clin North Am.

[5] Ashley M, Holden V (1998). An immediate adhesive bridge using the
natural tooth. Br Dent J.

[6] Chafaie A, Portier R (2004). Anterior Fiber-reinforced Composite Resin
Bridge: A Case Report. Pediatr Dent.

